# Exploring Mitochondrial Evolutionary Pathways: Insights into the Origin of the Endemic Ohrid Trout

**DOI:** 10.3390/life15010052

**Published:** 2025-01-03

**Authors:** Anila Hoda, Chiara Locci, Ilenia Azzena, Noemi Pascale, Ilaria Deplano, Roland Kristo, Arian Demiri, Fabio Scarpa, Marco Casu, Daria Sanna

**Affiliations:** 1 Department of Animal Sciences, Agricultural University of Tirana, Kodër Kamëz, 1029 Tirana, Albania; ahoda@ubt.edu.al; 2 Department of Biomedical Sciences, University of Sassari, Viale San Pietro 43b, 07100 Sassari, Italy; c.locci3@phd.uniss.it (C.L.); npascale@uniss.it (N.P.); i.deplano@phd.uniss.it (I.D.); fscarpa@uniss.it (F.S.); 3 Department of Veterinary Medicine, University of Sassari, Via Vienna 2, 07100 Sassari, Italy; iazzena@uniss.it (I.A.); marcasu@uniss.it (M.C.); 4 Department of Chemical, Physical, Mathematical, and Natural Sciences, University of Sassari, Via Vienna 2, 07100 Sassari, Italy; 5 Ministry of Agriculture and Rural Development, Blvd Deshmoret e Kombit, Nr. 2, 1001 Tirana, Albania; roland.kristo@bujqesia.gov.al; 6 Directory of Fishery and Aquaculture Services, Blvd Deshmoret e Kombit, Nr. 2, 1001 Tirana, Albania; arjan.demiri@dshpa.gov.al

**Keywords:** Lake Ohrid, Korani trout, mitochondrial DNA, Control region, *Salmo l. typicus*, *Salmo l. aestivalis*, *Salmo l. lumi*, *Salmo l. balcanicus*

## Abstract

The Ohrid trout, *Salmo letnica*, is an endemic species of Lake Ohrid, one of Europe’s oldest lakes, located on the Albania-North Macedonia border. This species exhibits distinct morphotypes—*Salmo letnica typicus*, *Salmo letnica aestivalis*, *Salmo letnica balcanicus*, and *Salmo letnica lumi*—that differ in morphology and spawning behaviour. However, the extent of their genetic differentiation remains unclear. This study aimed to investigate the genetic variability and population structure of *Salmo letnica* morphotypes using the mitochondrial Control Region as molecular marker. We obtained 127 sequences from *Salmo letnica* morphotypes and compared them with sequences from other species within the genus *Salmo*. Phylogenetic and clustering analyses revealed no significant genetic structuring among the four morphotypes, suggesting an ecological differentiation not (yet) fixed at mitochondrial level. Additionally, our findings suggest that the modern *Salmo letnica* population likely originated in Lake Ohrid from *Salmo farioides* founders through evolutionary differentiation, potentially driven by environmental changes. Future studies incorporating a larger number of samples from both *Salmo letnica* and *Salmo farioides* are essential to fully understand the evolutionary and ecological dynamics of *Salmo letnica* morphotypes.

## 1. Introduction

Lake Ohrid, nestled among the Dassaretes Lakes in the Balkans (central Europe), holds a distinguished status as one of Europe’s oldest bodies of water, with estimates of its age ranging between two and four million years old [1]. The Dassaretes Lakes consist of a group of lakes that includes Lake Ohrid (Albania, North Macedonia), Lake Prespa (Albania, Greece, North Macedonia), Lake Mikri Prespa (Albania, Greece,), and Lake Maliq (Albania) [2]. Lake Ohrid, positioned at an altitude of 693 m [3], is the most renowned among these lakes, with a watershed extending over 1000 km² [4]. Moreover, it ultimately drains into the Adriatic Sea via the Drim River [4]. Remarkably, Lake Ohrid’s maximum water depth of 288 m and an average depth of 155 m [5] have shielded it from glaciation, setting it apart as a geological marvel [1]. The lake benefits from a steady influx of water from karstic surface and sub lacustrine springs, ensuring a relatively stable water level and fostering a habitat conducive to a high degree of endemism [1].

Predominantly fed by spring water, including contributions from neighboring Lake Prespa [6], Lake Ohrid maintains oligotrophic conditions characterized by high water clarity [3,5]. Since the early 20th century, the lake has been the subject of scientific scrutiny, with studies particularly focused on its endemic and relict species (e.g., [1,2,3,6,7,8]). The lake’s distinctive characteristics render it an invaluable natural laboratory for comprehending long-term environmental changes in the central northern Mediterranean region [3,9].

The enduring lineage of Lake Ohrid’s flora and fauna, preserved over millions of years, is a testament to its unique characteristics [1]. Approximately 40% of its fish species are endemic [2], underscoring its significance as a biodiversity hotspot. Among these are several endemic trout species, including the Ohrid trout, *Salmo letnica* Karaman, 1924, locally known as “Korani trout” [10], which stands out as a key subject for further investigation due to its distinct evolutionary adaptation (i.a., [11]). Additionally, another endemic trout species, *Salmo ohridanus* (Steindachner, 1892) [12,13], previously classified as *Acantholingua ohridana* (Hadžišče, 1961), has been identified in this lake [2].

Notably, the taxonomic classification and evolutionary history of the endemic trout species *Salmo letnica* remain subjects of ongoing debate. Initially described by Karaman (1924) [6,14], subsequent research by Stefanoviç (1948) delineated three distinct populations within the *letnica* species: *Salmo l. typicus, Salmo l. aestivalis,* and *Salmo l. balcanicus* [7]. These populations exhibit significant differences in various morphological traits and behaviors, including spawning preferences [7]. *Salmo l. typicus* population spawns in winter, specifically in January and February, in littoral and sublittoral areas, whereas *Salmo l. aestivalis* population prefers deeper rocky habitats during the summer months. In contrast, *Salmo l. balcanicus* population favors spawning near the lake outlet between December and January [7]. Additionally, two other trout populations have been identified in the Lake Ohrid area: *Salmo l. lumi* Poljakov, Filipi & Basho, 1958 and *Salmo farioides* Karaman, 1938 [15]. *Salmo l. lumi*, a river spawner, was found along the northern and western shores of the lake. Meanwhile, *Salmo farioides* has been detected in the smaller tributaries along the eastern lakeshore [16,17].

In more recent times, Rakaj and Flloko (1995) provided a detailed morphological description of the *Salmo letnica* (species) group, highlighting distinct intraspecies characteristics, especially between males and females [10]. Additionally, these authors detected a more pronounced differentiation of *Salmo l. lumi* in respect to the other three morphotypes of Korani trout, being distinguished by its longer, more robust body with distinct black and red spots [10].

*Salmo letnica* plays a significant role in Lake Ohrid’s ecosystem, constituting a substantial portion of salmonid catches, with efforts in artificial reproduction dating back to the 1950s [10]. However, over the past few decades, environmental changes and human pressures have significantly affected its populations. Currently, only two forms, *Salmo l. typicus* and *Salmo l. aestivalis*, are definitively observed. However, *Salmo l. lumi* and *Salmo l. balcanicus* still exist but are possibly dwindling in population [17]. This decline evidences the urgent need for targeted conservation efforts.

In response to these challenges, Kostoski et al. (2010) emphasized the importance of immediate conservation efforts to protect freshwater resources globally, particularly ancient lakes like Lake Ohrid [9]. This lake is increasingly threatened by anthropogenic pressures, non-native species, and climate change. To mitigate these threats, effective conservation strategies should primarily focus on watershed management and establishing conservation and coastal zone management areas to safeguard the lake’s unique ecosystem [9].

It is important to note that this area requires sustainable conservation and management strategies, as highlighted by Smederevac-Lalić et al. (2023) [18]. Indeed, in the Western Balkans, balancing agricultural modernization with environmental sustainability remains challenging, as economic priorities frequently overshadow ecological concerns. The fisheries sector, essential for both food security and income, suffers from weak organization and insufficient enforcement, leading to unsustainable practices [18].

In this context, the Lake Ohrid Conservation Project (LOCP), launched in the late 1990s, represents a collaborative effort between Albania and Macedonia, aimed at jointly managing the lake’s watershed to preserve its biodiversity [19].

Recent studies have further highlighted the decline in the trout population in Lake Ohrid, reinforcing the need for coordinated management efforts to ensure the fishery’s sustainability [20,21]. Additionally, understanding the environmental factors influencing the growth and development of *Salmo letnica* is essential for its long-term conservation [22].

From a conservation perspective, studies specifically investigating the genetic structure and evolutionary history of Ohrid trout are limited. Most research has focused primarily on mitochondrial DNA analyses [13,17,23,24,25,26,27,28]. These studies have consistently placed *Salmo letnica* within the Adriatic phylogeographical lineage of brown trout (*Salmo trutta*) [13,17,27]. Efforts have also been made to explore the historical demography of Ohrid trout, with a particular focus on the genetic differentiation between its winter (*Salmo l. typicus*) and summer (*Salmo l. aestivalis*) spawning forms. This distinction is crucial due to the unique genetic makeup and ecological significance of *Salmo letnica* [23,25]. For instance, Sušnik et al. (2007) conducted a study using both mitochondrial DNA and microsatellites as molecular markers to investigate the genetic structuring between these two forms [26]. Their results did not reveal significant genetic differentiation within the species [26]. These results, however, contrasted with the findings of Sell and Spirkovski (2004), who reported a high degree of genetic differentiation between the winter and summer spawning forms of Ohrid trout [17]. The discrepancy was later explained by Sušnik et al. (2006, 2007), who attributed the variation observed in that earlier study to instability at the 3′ end of the mitochondrial Control Region. This instability, caused by intramolecular mechanisms, was deemed unreliable for phylogenetic investigations [13,26].

The limited availability of genetic data and the possible ambiguity in previous findings underscore the need for more detailed genetic research to clarify the extent of differentiation within the species and to better understand its genetic variability and evolutionary origin.

In light of these concerns, this study aimed to investigate the genetic variability and potential genetic structuring of the four distinct morphotypes of the Ohrid trout (*Salmo letnica*) population through mitochondrial genotyping. To achieve this, a portion of the mitochondrial Control Region was employed as molecular marker. This non-coding region was chosen due to its general high variability and neutrality to selective pressures, making it effective in detecting genetic divergence due to distinct phylogenetic origins. This region is recognized for its utility in assessing genetic structure within populations and identifying interspecific differences [29,30]. Additionally, the availability of numerous sequences from this mitochondrial region in the GenBank database, associated with selected *Salmo* species, enabled an examination of the genetic relationships and a comparison between the Lake Ohrid trout obtained in the present study and other trout species inhabiting or observed in the lake. This analysis sought to shed light on the evolutionary history of *Salmo letnica* and provide actionable data essential for effective conservation strategies aimed at preserving the biodiversity of Lake Ohrid.

## 2. Materials and Methods

### 2.1. Sample Collection

Ohrid Trout (*Salmo letnica*) is a commercial species fished in both Albania and North Macedonia. In Albania, fishing is conducted by the Fishermen’s Association through a co-management process, while in North Macedonia, it is managed via concession agreements. Listed as “Endangered” on the IUCN (International Union for Conservation of Nature) Red List of Threatened Species [31], Ohrid trout is subject to strict fishing regulations: minimum landing sizes (32 cm in Albania and 35 cm in North Macedonia) and designated closed fishing seasons (December 1 to February 28 in Albania, and December 1 to March 20 in North Macedonia) (Albanian legislation: https://qbz.gov.al/eli/urdher/2022/03/31/149/cc5c689b-5245-417a-978d-52346bbb2543, accessed on 1 August 2022; North Macedonian legislation: https://mzsv.gov.mk, accessed on 1 August 2022).

**Figure 1:**
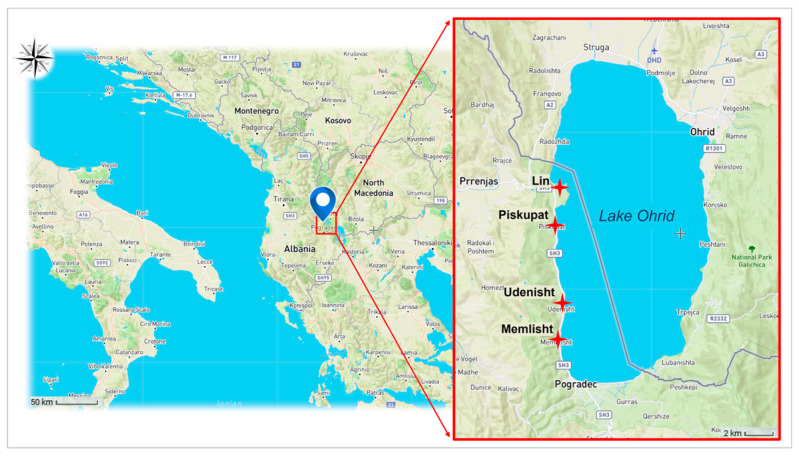
Sampling locations of Ohrid brown trout (*Salmo letnica*). The map highlights the four sampling sites (marked in red) where the Control Region sequences analyzed in the current study were obtained.

In the present study, samples of Ohrid trout were collected from multiple locations within the Albanian side of Lake Ohrid between September 2022 and July 2023, excluding the closed fishing season previously mentioned (Figure 1 and Supplementary Table S1), representing four distinct morphological forms: *typicus*, *balcanicus*, *lumi* and *aestivalis*. A total of 127 individuals were included in the analyses, comprising 35 specimens of *Salmo l. typicus*, 36 specimens of *Salmo l. balcanicus*, 22 specimens of *Salmo l. lumi*, and 34 specimens of *Salmo l. aestivalis*.

The sampling process was particularly time-intensive due to the rarity of certain forms, notably *Salmo l. lumi*. While *Salmo l. lumi* is not extinct, it is exceedingly rare, which made it challenging to reach the desirable target of 30 specimens for this form. During some expeditions, only 1–2 specimens were found, while on other occasions, none were located. This rarity significantly extended the sampling period, requiring nearly a year to complete. Despite these challenges, we aimed to achieve the required sample size of 30 individuals per morphological form of *Salmo letnica*, though this was difficult for the rarer forms.

All samples were obtained exclusively from deceased animals destined for the fish market upon the return of fishermen from fishing expeditions. Local members of the Fishermen’s Association facilitated access to licensed fishermen and boats specializing in Ohrid trout fishing, both for family consumption and commercial purposes. These professionals, who have studied *Aquaculture and Fishery*, are also involved in operations at the Koran Restocking Centre in Lin.

Their logistical support was essential. Indeed, at designated shore centers (Lin, Piskupat, Udenisht, and Memlisht), they assisted in distinguishing the four forms based on their morphological characteristics and supported the separation of samples for subsequent molecular analyses. Additionally, specimens were collected by taking fin clips from each individual.

The fin clips were preserved in absolute ethanol and subsequently utilized for DNA extraction.

### 2.2. Diagnostic Molecular Analyses

Total genomic DNA was extracted from a portion of fin tissue using the Mackerey-Nagel NucleoSpin Tissue Kit (MACHEREY-NAGEL GmbH and Co. KG, Duren, Germany), following the manufacturer’s protocol. DNA quantification was carried out with the Nanodrop™ Lite Spectrophotometer (Thermo Scientific; Waltham, MA, USA), yielding an average concentration of 80 ng/μL. A portion of the mitochondrial Control Region was amplified by standard PCR using the following primers: L19CR (forward) (5′-CCACTAGCTCCCAAAGCTA-3′) [23] and K-Rev CR (reverse) ((5′-CAGGACCAAGCTTTTGTGCTTACG-3′) [32]. The PCR reactions were prepared in a total volume of 25 μL, containing approximately 10 ng of genomic DNA, 0.6 μM of each primer, and a PuReTaq Ready-To-Go PCR bead (GE Healthcare, Wauwatosa, WI, USA), which included stabilizers, bovine serum albumin (BSA), deoxynucleotide triphosphates, 2.5 units PuReTaq DNA polymerase, and buffer. Upon reconstitution to a final volume of 25 μL, the dNTP and MgCl2 concentrations were set to 200 μM and 1.5 mM, respectively. PCR cycles were performed in a GeneAmp PCR System 9700 Thermal Cycler (Applied Biosystems, Waltham, MA, USA), setting the thermal cycling conditions as follows: an initial denaturation at 94 °C for 4 min, followed by 35 cycles of 30 s at 94 °C, 30 s at 51 °C, and 30 s at 72 °C, with a final extension step of 10 min at 72 °C and cooling at 4 °C. To ensure the reliability of the PCR and to verify the absence of contamination, positive controls (high-quality DNA) and negative controls were also included in the analysis. The PCR products were analyzed through electrophoresis on 2% agarose gels prepared in 1x TAE buffer (Tris-Acetate-EDTA, pH 8.3) and stained with Gel Red Nucleic Acid Stain (Biotium Inc., Fremont, CA, USA). Purification of the PCR products was performed using ExoSAP-IT (USB Corporation, Cleveland, OH, USA). Both forward and reverse strands were sequenced using the same primers as in the PCR, with sequencing performed by an external core service (Macrogen Europe, Milan, Italy).

### 2.3. Phylogeographic and Phylogenetic Analysis

A total of 127 Control Region sequences (553 bp) from Ohrid trout individuals were obtained in the present study (Supplementary Table S1) and have been deposited in the GenBank online database (Accession numbers PQ583195–PQ583321).

All newly obtained sequences were verified through BLAST analysis on the GenBank nucleotide database (www.ncbi.nlm.nih.gov, accessed on 6 June 2024) and confirmed to belong to the species *Salmo letnica*, with a 100% identity match.

Sequences were aligned using the Clustal Omega algorithm, implemented in the MPI Bioinformatics Toolkit (available at https://toolkit.tuebingen.mpg.de/tools/hhpred, accessed on 6 June 2024). The alignment was manually checked and verified for the occurrence of polymorphisms with Unipro UGENE v.35 [33].

The new sequences obtained in the present study were merged with all available sequences from the GenBank database for *Salmo* species (*Salmo letnica*, *Salmo l. lumi*, *Salmo ohridanus*) inhabiting Lake Ohrid. This integration was based on prior studies documenting these species in the area [13,26,28,34,35]. Additionally, sequences of *Salmo farioides* from the White Drin River in Albania [36] were included (see Supplementary Table S2 for details). Hence, this comprehensive dataset, encompassing both newly generated and publicly available sequences, consisted of a total of 164 sequences.

To enhance our understanding of the relationship between our samples and the major brown trout (*Salmo trutta*) phylogeographical lineages (AT—Atlantic, AD—Adriatic, ME—Mediterranean, MA—Marmoratus, DA—Danubian) [37,38,39,40,41], five representative sequences were incorporated into the whole dataset (see Supplementary Table S2 for details). Furthermore, a sequence belonging to *Salmo salar* species was used as outgroup.

The genetic diversity among sequences was evaluated by calculating several key parameters, including the number of polymorphic sites (S), number of haplotypes (H), nucleotide diversity (π), and haplotype diversity (h). This analysis was carried out using the DnaSP 6.12.03 software package (developed by Universitat de Barcelona, Barcelona, Spain) [42].

The best probabilistic model for sequence evolution was identified using jModeltest 2.1.1 [43] via a maximum likelihood optimized search, evaluated by the Akaike (AIC) and Bayesian Information Criterion (BIC). Both criteria identified the GTR + I + G model as the best fit for the whole dataset.

Evolutionary relationships among haplotypes and taxonomic entities were examined by using MrBayes 3.2.7 [44]. The parameters for the analysis were set as follows: NST = 6, rates = invgamma, ngammacat = 4. The procedure included two independent runs of four Metropolis-coupled Markov-chain Monte Carlo (MCMCMC) simulations (one cold and three heated chains) for 5,000,000 generations, sampling trees every 1000 generations. The first 25% of the 10,000 sampled trees were discarded as burn-in. Nodes with a posterior probability above 0.9 were considered statistically well supported. Convergence of the chains was tested by verifying that the Average Standard Deviation of Split Frequencies (ASDSF) approached zero, following Ronquist et al. [44], and that the Potential Scale Reduction Factor (PSRF) was approximately equal to 1, as suggested by Gelman and Rubin [45]. The resulting phylogenetic tree was edited and visualized using FigTree 1.4.0 (available online: http://tree.bio.ed.ac.uk/software/figtree/, accessed on 15 July 2024).

To identify potential subgroups within genetic clusters and assess genetic variability among sequences, we conducted a principal coordinate analysis (PCoA) using GenAlEx 6.5 [46]. This PCoA reconstruction was based on a pairwise p-distance matrix of genetic data, aiming to examine the dissimilarity represented by the genetic variability among the analyzed sequences. This analysis was performed on both the entire dataset and a subset, based on the results obtained from the initial run.

A median-joining network [47] was constructed using the Network 10.2.0.0 software package (available at www.fluxus-engineering.com, accessed on 6 June 2024) from Colchester, UK. This network was generated to analyze the genetic relationships among haplotypes and to identify potential discrete genetic clusters. In this analysis, both transitions and transversions were weighted equally. Due to uncertainties surrounding the occurrence of retromutation events, each observed polymorphism was assigned an equal weight of 10.

## 3. Results

A total of 6 polymorphic sites were detected among the 127 analyzed sequences, resulting in 6 distinct Control Region haplotypes (see Table 1). Moreover, reduced and comparable genetic diversity estimates were observed across the four forms, except for *Salmo letnica lumi*, which showed relatively lower nucleotide divergence compared to the other morphotypes, specifically with a nucleotide diversity an order of magnitude lower.

**Figure 2:**
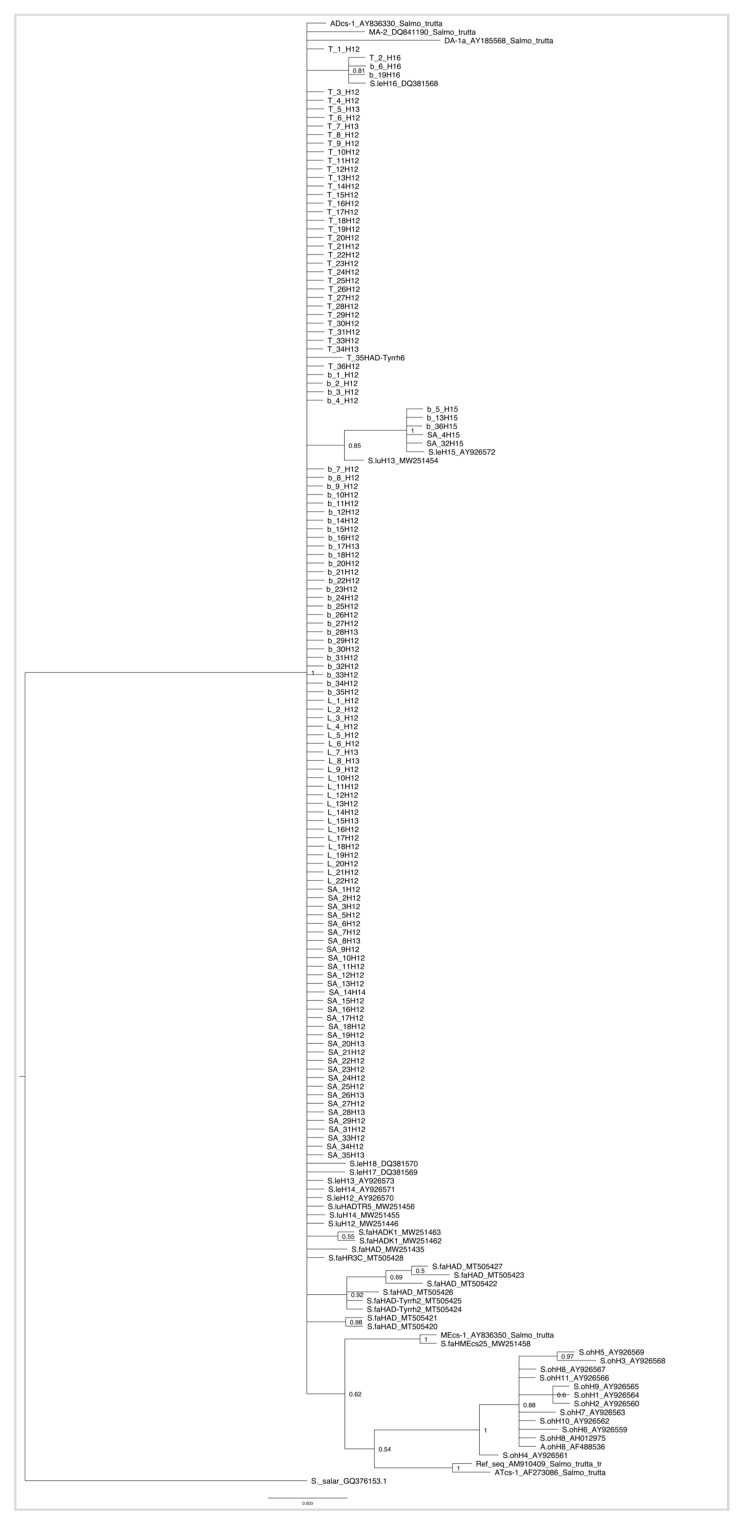
Bayesian phylogenetic tree based on the complete dataset, including *Salmo letnica* sequences and selected *Salmo* species. Specimens from the present study are labelled according to their species abbreviations: “T” for *Salmo l. typicus*, “b” for *Salmo l. balcanicus*, “L” for *Salmo l. lumi*, and “SA” for *Salmo l. aestivalis*, followed by their corresponding assigned haplotype (“H”). GenBank sequences are labelled using species abbreviations: “S.le” for *Salmo letnica*, “S.lu” for *Salmo l. lumi*, “S.fa” for *Salmo farioides*, and “S.oh” for *Salmo ohridanus*, followed by their respective assigned haplotype and GenBank accession numbers. *Salmo trutta* sequences are labelled with the assigned haplotype, GenBank accession number, and species name. Node values represent posterior probabilities. In general, support values exceeding 0.9 were considered as well supported.

The Bayesian evolutionary tree (Figure 2), derived from a dataset comprising both newly sequenced samples and all relevant sequences of specimens belonging to the genus *Salmo*, displayed well-supported branches with a posterior probability of 1 at the major node. Overall, the tree revealed a statistically significant lack of genetic structure based on taxonomic entities. With midpoint rooting, it correctly placed *Salmo salar* as the outgroup, positioned externally to the main clade. The main clade was further structured into several sub-clades, forming two primary groups. The first group consisted of *Salmo ohridanus*, which stood out as an exception to the general lack of taxonomic structuring; this species clustered outside the most inclusive clade and was characterized by 11 haplotypes. The second group, moving inward through the tree, represented the most inclusive clade. This large polytomy encompassed *Salmo letnica*, *Salmo l. typicus*, *Salmo l. balcanicus*, *Salmo l. lumi*, *Salmo l. aestivalis*, and *Salmo farioides*. This clade constituted an heterogenous non-monophyletic group, that, based on the results, was at least polyphyletic. Notably, the phylogenetic tree did not reveal a division consistent with the taxonomic framework to create discrete groups corresponding to the number of species included. All the sub-clades within this clade comprised multiple *Salmo* species. Interestingly, a fully supported sub-clade included *Salmo letnica* (AY926572), *Salmo l. balcanicus* (b_5, b_13, b_36), and *Salmo l. aestivalis* (SA_4, SA_32).

The absence of genetic structuring based on taxonomic criteria was also confirmed by the Principal Coordinates Analysis (PCoA, Supplementary Figure S1a), which accounted for 93.71% of the genetic variability in the dataset. The graph showed that *Salmo ohridanus* was separated from the other terminals, appearing as the most heterogenous and variable among those included in the analyses. Moreover, the PCoA revealed the occurrence of two main genetic groups: Group 1, comprising *Salmo farioides* (MW251458, MT505423), *Salmo letnica* (AY926572), *Salmo l. balcanicus* (b_5, b_13, b_36), and *Salmo l. aestivalis* (SA_4, SA_32), and Group 2, a more inclusive group, containing the remaining terminals belonging to the species *Salmo letnica*, *Salmo farioides*, *Salmo l. typicus*, *Salmo l. balcanicus*, *Salmo l. lumi*, and *Salmo l. aestivalis*. Notably, Group 1 was partially consistent with the fully supported sub-clade observed in the phylogenetic tree. However, while the sub-clade did not include *Salmo farioides*, the PCoA grouped a few *Salmo farioides* sequences with *Salmo letnica*, *Salmo l. balcanicus*, and *Salmo l. aestivalis*. This suggests a slight discrepancy between the results of the phylogenetic analysis and the PCoA, highlighting differences in how these analyses represent the genetic relationships of *Salmo farioides*, along with *Salmo letnica* species. This difference could be the consequence of methodological variations between the two approaches (phylogenetic tree vs. PCoA), which base their results on evolutionary divergences, along with genetic differences, versus general similarities, respectively.

In particular, the phylogenetic tree is based on evolutionary models and evidences divergences driven by specific mutations accumulated over time. This branching structure reflects the evolutionary history of the species and highlights ancestral relationships. In contrast, PCoA is based on a dissimilarity matrix in which samples are distributed across a multidimensional space according to genetic similarities among sequences, without considering ancestral relationships. For this reason, in PCoA, genetically similar samples may appear close in space even if they do not share a recent common ancestor and are not phylogenetically related.

Additionally, phylogenetic trees are sensitive to mutations that are significant for lineage distinctions, especially recent ones, whereas PCoA considers only overall genetic distance, ignoring the temporal sequence of mutations.

Group 2 was further investigated with an additional PCoA (Supplementary Figure S1b), which explains 75.65% of the genetic variability within this subset. The graph revealed several outliers: a few *Salmo farioides*, one *Salmo letnica typicus* (T_35), and one *Salmo l. lumi* (MW251454). The analysis also identified two genetic groups labelled as A and B. Group A included *Salmo l. lumi* (MW251455, L_7, L_8, L_15), *Salmo letnica* (AY926571, AY926573), *Salmo l. typicus* (T_5, T_7, T_34), *Salmo l. balcanicus* (b_17, b_28), and *Salmo l. aestivalis* (SA_8, SA_14, SA_20, SA_26, SA_28, SA_35). Group B encompassed all remaining terminals of *Salmo letnica*, *Salmo l. lumi*, *Salmo l. aestivalis*, *Salmo l. balcanicus*, and *Salmo l. typicus*. In general, the PCoA did not reveal any genetic structuring corresponding to the subdivision into morphotypes.

**Figure 3:**
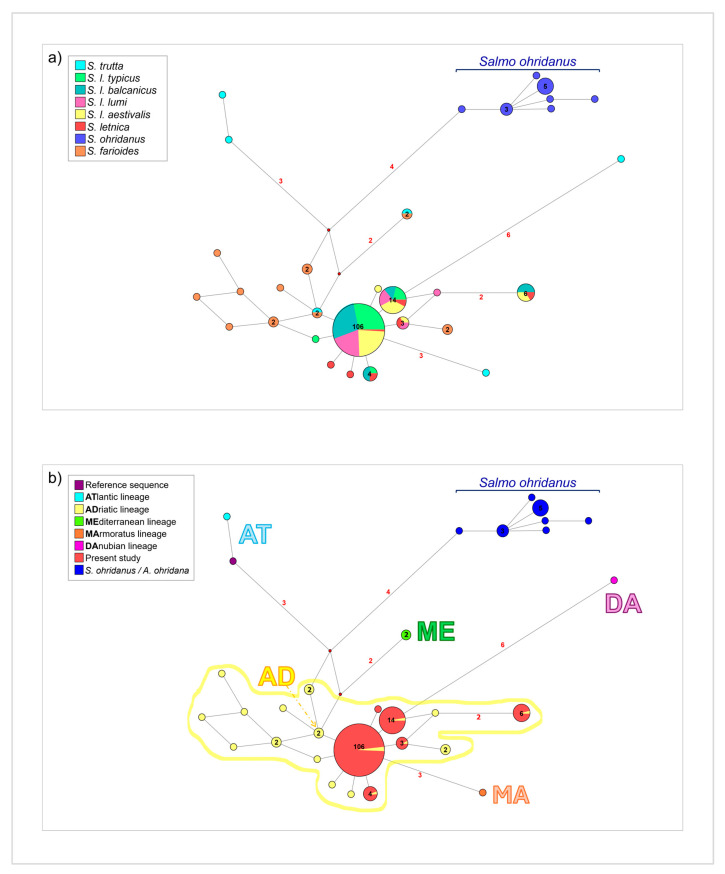
Network (Median-Joining) analysis of the whole dataset, including *Salmo letnica* sequences and selected *Salmo* species. The network is represented based on two categories: (**a**) *Salmo* species and (**b**) major phylogeographical lineages of brown trout (*Salmo trutta*). Each circle represents a unique haplotype, with its diameter proportional to the haplotype frequency. The number of mutations greater than 1 is indicated along the branches. Small red circles represent median vectors, which correspond to intermediate missing or unsampled haplotypes. Colors in the network indicate the *Salmo* species (**a**) and the main phylogeographical lineages of brown trout (*Salmo trutta*) (**b**), as shown in the legend. The number of individuals sharing the same haplotype, when the frequency exceeds 1, is displayed inside the circle.

The network analysis of the whole dataset (Figure 3) was represented according to two categories: *Salmo* species (a) and major brown trout (*Salmo trutta*) phylogeographical lineages (b). In Figure 3a, three principal haplotypes were identified, predominantly including *Salmo letnica* species. The first and most common haplotype encompassed 106 individuals, comprising approximately 85% of *Salmo l. typicus*, 80% of *Salmo l. balcanicus*, 81% of *Salmo l. lumi*, 73% of *Salmo l. aestivalis*, and 1 *Salmo l.* sequence representing haplotype 12 (AY926570). This haplotype showed an incipient star-like shape, indicating a marked founder effect. It was surrounded by several derived haplotypes, differing by a few mutations, which mostly represented *Salmo farioides* sequences. Furthermore, 3 and 6 to 7-point mutations away from this haplotype were 3 private haplotypes belonging to *Salmo trutta*. The second most frequent haplotype, separated from the first by a single point mutation, contained 9% of *Salmo l. typicus*, 6% of *Salmo l. balcanicus*, 14% of *Salmo l.a lumi*, 15% of *Salmo l. aestivalis*, and 1 *Salmo letnica* sequence corresponding to haplotype 13 (AY926573). Moreover, a single *Salmo trutta* sequence diverged from this haplotype 6-point mutations. The third haplotype, which was 3-point mutation away from the second, comprised 8% of *Salmo l. balcanicus*, 6% of *Salmo l. aestivalis*, and a sequence of *Salmo l.* representing haplotype 15 (AY926572). Additionally, all sequences belonging to *Salmo ohridanus* diverged by 7-point mutations from the most common lineage, forming a separate cluster characterized by one common haplotype with six derived ones. This was consistent with findings from the phylogenetic tree and the PCoA analyses. In Figure 3b, the majority of *Salmo farioides* sequences, along with all *Salmo letnica* sequences, clustered within the Adriatic lineage (AD). This large haplogroup was surrounded by the other selected brown trout lineages (Atlantic, Mediterranean, Danubian, Marmoratus). Notably, as previously reported, *Salmo ohridanus* formed a separate group, distinct from all recognized lineages in the analysis.

## 4. Discussion

The present study represents a preliminary investigation that provides insights into the genetic diversity of the endemic *Salmo letnica* in Lake Ohrid, using the mitochondrial Control Region as molecular marker. This region, widely used in genetic diversity studies, was chosen because it was the only molecular marker that permitted an extensive comparison with all the molecular data already available in literature. Moreover, it is particularly valuable for identifying incipient genetic structuring within populations and for detecting interspecific divergence [30,48].

Indeed, in this study, mitochondrial DNA has been effective in detecting genetic divergence between *Salmo letnica* and its sympatric congener, *Salmo ohridanus*. The latter species consistently exhibited a relevant level of genetic differentiation, forming a distinct cluster in all the analyses performed. This outcome aligns with previous studies, further supporting the genetic divergence of *Salmo ohridanus* from its related species [13,26,27]. It is likely that these two species have coexisted in Lake Ohrid over time, evolving independently due to differences in their ecological niche and reproductive behaviors [13,33]. The exact timing of *Salmo ohridanus* colonization of the lake, as well as its morphological divergence from *Salmo letnica* (Ohrid trout), remain unclear [13]. Interestingly, the distinct taxonomic position of *Salmo ohridanus* is attributed to its closer genetic relationship with *Salmo obtusirostris* [27], a species found in the Dalmatian River systems, which has been identified as its sister taxon [13,26,34]. The Dalmatian River systems are located in Croatia along the Adriatic coast and include the Neretva, Cetina, Krka, and Zrmanja rivers. These rivers are situated quite a distance from Lake Ohrid, which lies within a separate watershed in the western Balkans. Of note, Lake Ohrid’s basin is distinct from the Adriatic drainage basin that encompasses the Dalmatian rivers.

Interestingly, despite the use of mitochondrial DNA as marker, our findings underscored a general genetic homogeneity within the different *Salmo letnica* populations analyzed, with no distinctive genetic traits among the *Salmo letnica* morphotypes (*Salmo l. typicus*, *Salmo l. balcanicus*, *Salmo l. lumi*, *Salmo l. aestivalis*), as revealed by both clustering and phylogenetic analyses. Indeed, the four morphotypes, which are in general associated with ecological adaptation phenomena [7], did not show relevant nucleotide differences. This lack of genetic divergence is consistent with results reported by Sušnik et al. (2007), who did not report the presence of population sub-structuring within the lake using both nuclear and mitochondrial markers. This occurrence suggests a potential common origin of Ohrid trout mitochondrial lineages, likely due to a founder effect that characterized the ancestral population in Lake Ohrid [26].

During the first half of the twentieth century, two main hypotheses have been proposed to explain the origin of Ohrid trout morphological forms. Stefanoviç (1948) suggested that these morphotypes evolved through intralacustrine speciation [7], while Karaman (1957) proposed independent invasions by distinct species [49].

Our results support the first hypothesis. While we acknowledge that the use of a single DNA fragment in our analyses might have influenced the results, and that informative genetic variation might have exclusively involved coding regions of the genome, a plausible scenario is that the adaptive processes leading to the emergence of the four *Salmo letnica* morphotypes may have been driven by differential gene expression in mitochondrial or nuclear DNA, possibly boosted by environmental pressures. This hypothesis warrants further investigation to validate its potential role in shaping the observed patterns. However, it should also be considered that differential gene expression, eventually prompted by epigenetic mechanisms, could result in morphological and ecological differences, producing phenotypic variation without altering DNA sequences.

Our findings may challenge the current taxonomic status of *Salmo letnica*, indicating that the adaptive processes shaping the ecological differentiation of the four Ohrid trout morphological forms may not be mirrored in their mitochondrial DNA. Similarly, two endemic species of the genus *Champsochromis* (Vertebrata, Perciformes, Cichlidae) inhabiting Lake Malawi exhibit differences in the morphology but show no mitochondrial genetic diversity [48]. This phenomenon has been associated with the absence of strong reproductive barriers and recent speciation, with morphological changes reflecting evolutionary processes while genetic differentiation remains limited. This highlights that environmental factors, such as habitat specializations or diet, can drive morphological diversification without corresponding changes in mitochondrial genetic structure [48].

Notably, ancient lakes may allow existing species to evolve through intralacustrine speciation, while also functioning as evolutionary reservoirs [2,50,51]. This variation suggests that intralacustrine speciation plays a significant role in diversity processes [2]. Furthermore, the processes of speciation could be influenced by varying degrees of isolation within the Ohrid watershed, potentially caused by intralacustrine barriers existing in both vertical and horizontal dimensions [2,52]. For instance, several morphological forms of the endemic *Proasellus* species (Crustacea, Isopoda, Asellidae) have been observed in Lake Ohrid, each associated with different bathymetric zones of the lake. Accordingly, the development of the four morphotypes of Ohrid trout may be a response to the different environmental features of the lake [2]. A similar case of intralacustrine diversification is seen in the Caucasian Lake Sevan, where the Sevan trout, *Salmo ischchan* Kessler, 1877, has differentiated into four sympatric ecomorphs or species [11]. These morphological forms are each associated with distinct spawning resources, showing how resource partitioning within a single lake system may drive speciation and contribute to ecological diversity [11].

Our hypothesis is further supported by the case of lake char, *Salvelinus namaycush* (Walbaum, 1792), in Lake Superior (North America) [53]. In this species, four morphologically distinct ecotypes showed no significant genetic structuring. This suggests that the species may be in an early stage of speciation, where phenotypic differences are associated with ecological characteristics, such as water depth, and potentially driven by diversifying selection acting on adaptive genes [53]. Likewise, *Salmo letnica* in Lake Ohrid may exhibit similar adaptive processes, with phenotypic variation likely emerging due to ecological factors despite limited genetic differentiation. Future studies are needed, incorporating additional molecular markers, particularly nuclear DNA, alongside mitochondrial DNA to validate this hypothesis. Such studies could also further explore the ecological divergence and niche specialization of Ohrid trout in relation to environmental gradients within the lake. This could offer valuable insights into the mechanisms underlying speciation in this ecosystem.

Another important outcome is that the network analysis clustered *Salmo letnica* and *Salmo farioides* sequences within the brown trout’s Adriatic lineage, consistent with the findings of Snoj et al. (2009) [27]. According to Bianco (2014), *Salmo farioides* would represent the native population of the Adriatic trout lineage [54]. Commonly known as the brook-dwelling trout, *Salmo farioides* inhabits streams ranging from the Krka to the Drim–Skadar River systems [55]. The Drim River has two main branches: the Black Drim (Crni Drim), which originates from Lake Ohrid and flows through North Macedonia into Albania, and the White Drim (Beli Drim), which begins in Kosovo. These branches merge in northern Albania, forming the Drim River, which ultimately flows into the Adriatic Sea [55]. Historically, prior to the construction of hydropower dams in 1960s [27], Lake Ohrid was naturally connected to the Drim River, creating a continuous aquatic network that spatially connected the two species. However, in 1969, the Spilje Hydropower Plant was activated [56], likely disrupting the direct connection between Lake Ohrid and the Drim River. This interruption may have played a role in isolating *Salmo letnica* and *Salmo farioides*, contributing to their independent evolutionary paths.

Additionally, the absence of haplotype sharing between *Salmo letnica* and *Salmo farioides* suggests that the present-day *Salmo letnica* populations likely evolved from a small group of *Salmo farioides* founders that became landlocked in Lake Ohrid as a consequence of the dam construction. Over time, evolutionary forces, such as genetic drift first and selective sweep second, may have led to the specialization and adaptation of this founding population to the environmental conditions of Lake Ohrid, resulting into the modern *Salmo letnica* population, while *Salmo farioides* continued evolving in its riverine habitat. This phenomenon aligns with adaptive mechanisms of those allochthonous species in which haplotypes that are common in the derivative population are rare in the source population. Such a pattern was also observed for the alien invasive species *Fistularia commersonii*, *Procambarus virginalis* and *Callinectes sapidus* [57,58,59].

This scenario can be further explained by the punctuated equilibrium model, which proposes that species experience extended periods of relative evolutionary stability, followed by brief intervals of rapid evolutionary change and speciation, often prompted by radical environmental shifts [60,61]. The isolation of *Salmo farioides* in Lake Ohrid, following the damming of the Drim river, may have led the species to undergo a rapid evolutionary adaptation, accounting for the differentiation into the contemporary *Salmo letnica*. This scenario was potentially followed by the ecological adaptation of Ohrid trout to the lake’s environmental conditions, which may have contributed to the emergence of the four *Salmo letnica* morphotypes. These morphological forms may currently be in the early stages of an incipient speciation process, which might not yet have produced detectable genetic differentiation (at least at the mitochondrial Control Region) within the species.

## 5. Conclusions

This pilot study provides valuable hints for future inferences on the complex dynamics between genetic variation and ecological differentiation in the endemic Ohrid trout, *Salmo letnica*. Despite the presence of distinct *Salmo letnica* morphotypes in Lake Ohrid, our analysis of the mitochondrial Control Region revealed no evidence of genetic structuring, suggesting a possible overall low genetic variability for the mitochondrial DNA. In this context, morphological variability may have been acquired as a consequence of rapid adaptation phenomena mediated by a reduced level of polymorphisms in coding regions. For this reason, the present morphotypes may represent ecologically adapted variants of *Salmo letnica* rather than genetically distinct subspecies. This interpretation suggests the potential adaptive ability of the Ohrid trout to the lake’s diverse environmental conditions.

Additionally, interesting results suggest that the modern *Salmo letnica* population of Lake Ohrid likely evolved from a few *Salmo farioides* founders, adapting to the lake’s environmental conditions over time after the interruption of the connection between Lake Ohrid and Drim River. This scenario emphasizes the role of adaptive evolution in shaping the biodiversity of Lake Ohrid. In contrast, the clear genetic divergence of *Salmo ohridanus* reinforces the distinct evolutionary trajectory of *Salmo letnica* within the lake.

Further investigations, integrating additional molecular markers, such as nuclear DNA, and, if possible, employing a genome-wide approach, are crucial to validate and support the results reported in this study. Detailed ecological studies are also essential to fully understand the drivers of phenotypic variation and speciation in the Ohrid Lake’s ancient ecosystem.

In particular, research involving a larger number of *Salmo farioides* and *Salmo letnica* individuals will be necessary to clarify their evolutionary relationship, contributing to a better comprehension of the origin of Ohrid trout and its morphotypes.

Finally, our findings may have significant implications for the management and conservation of Lake Ohrid’s ecological diversity, particularly in supporting efforts to protect its endemic trout species.

## Figures and Tables

**Table 1:** Sample sizes and genetic diversity estimates for the mitochondrial Control Region sequences obtained in this study, representing *Salmo letnica* individuals classified into four morphotypes. N: sample size; S: number of polymorphic sites; H: number of haplotypes; h: haplotype diversity; π: nucleotide diversity. Sites with gaps were not considered.

Sample	N	S	H	h	π
*Salmo l. typicus*	35	3	4	0.264	0.00050
*Salmo l. balcanicus*	36	5	4	0.348	0.00150
*Salmo l. lumi*	22	1	2	0.247	0.00045
*Salmo l. aestivalis*	34	4	4	0.401	0.00134
Whole dataset *	**127**	**6**	**6**	**0.319**	**0.00101**

* Total values for sample size and genetic diversity for the whole dataset, which includes all the *Salmo letnica* sequences obtained in the present study.

## Data Availability

Sequences obtained in the present study for the mitochondrial Control Region were deposited in the GenBank database under the accession numbers PQ583195–PQ583321.

## Supplementary Materials

The following supporting information can be downloaded at: https://www.mdpi.com/article/10.3390/life15010052/s1, Figure S1: Principal Coordinates Analysis (PCoA) performed on the dataset including *Salmo letnica* sequences and selected sequences belonging to *Salmo* species reported from Lake Ohrid. Graphs “a” and “b” are described in the text. The bi-dimensional plot illustrates genetic differentiation among samples based on the nucleotide substitutions per site within the dataset. Table S1: *Salmo letnica* samples collected in the present study. The collection period ranged from September 2022 to November 2022 and from March 2023 to July 2023. The table includes the sample code, GenBank accession number, sampling area and site, and species for each specimen; Table S2: Control Region Dataset. The table lists Control Region sequences belonging to *Salmo letnica* and other *Salmo* species which were used in the present study, sourced from the GenBank online database.

## References

[1] Stanković S. (1960). The Balkan Lake Ohrid and its living world. Monographiae Biologicae.

[2] Albrecht C., Wilke T. (2008). Ancient Lake Ohrid: Biodiversity and evolution. Hydrobiologia.

[3] Wagner B., Lotter A.F., Nowaczyk N., Reed J.M., Schwalb A., Sulpizio R., Valsecchi V., Wessels M., Zanchetta G. (2009). 40,000-year record of environmental change from ancient Lake Ohrid (Albania and Macedonia). J. Paleolimnol..

[4] Popovska C., Bonacci O. (2007). Basic data on the hydrology of Lakes Ohrid and Prespa. Hydrol. Process..

[5] Matzinger A., Spirkovski Z., Patceva S., Wüest A. (2006). Sensitivity of ancient Lake Ohrid to local anthropogenic impacts and global warming. J. Great Lakes Res..

[6] Karaman S. (1924). Pisces Macedoniae.

[7] Stefanoviç D. (1948). Recherches systematiques et écologiques sur les Salmonides d’Ohrid. Acad. Serbe Sci. Éd. Spéc..

[8] Wagner B., Wilke T., Francke A., Albrecht C., Baumgarten H., Bertini A., Combourieu-Nebout N., Cvetkoska A., D’Addabbo M., Donders T.H. (2017). The environmental and evolutionary history of Lake Ohrid (FYROM/Albania): Interim results from the SCOPSCO deep drilling project. Biogeosciences.

[9] Kostoski G., Albrecht C., Trajanovski S., Wilke T. (2010). A freshwater biodiversity hotspot under pressure—Assessing threats and identifying conservation needs for ancient Lake Ohrid. Biogeosciences.

[10] Rakaj N., Flloko A. (1995). Conservation status of freshwater fish of Albania. Biol. Conserv..

[11] Levin B., Simonov E., Gabrielyan B.K., Mayden R.L., Rastorguev S.M., Roubenyan H.R., Sharko F.S., Nedoluzhko A.V. (2022). Caucasian treasure: Genomics sheds light on the evolution of half-extinct Sevan trout, *Salmo ischchan*, species flock. Mol. Phylogenet. Evol..

[12] Phillips R.B., Matsuoka M.P., Konon I., Reed K.M. (2000). Phylogenetic Analysis of Mitochondrial and Nuclear Sequences Supports Inclusion of *Acantholingua ohridana* in the Genus *Salmo*. Copeia.

[13] Sušnik S., Knizhin I., Snoj A., Weiss S. (2006). Genetic and morphological characterization of a Lake Ohrid endemic, *Salmo* (*Acantholingua*) *ohridanus* with a comparison to sympatric *Salmo trutta*. J. Fish Biol..

[14] Karaman S. (1927). Salmonidi Balkana. Glas. Skopskog Naučn. Druš..

[15] Karaman S. (1938). Beitrag zur kenntnis der su¨sswasserfische Jugoslawiens. Bull. Soc. Sci. Skoplje.

[16] Kottelat M. (1997). European freshwater fishes. An heuristic checklist of the freshwater fishes of Europe (exclusive of former USSR), with an introduction for non-systematists and comments on nomenclature and conservation. Biologia.

[17] Sell J., Spirkovski Z. (2004). Mitochondrial DNA differentiation between two forms of trout *Salmo letnica*, endemic to the Balkan Lake Ohrid, reflects their reproductive isolation. Mol. Ecol..

[18] Smederevac-Lalić M., Špelić I., Đug S., Pengal P., Çobani M., Mrdak D., Piria M., Simić V., Simić S., Pešić V. (2023). Political and Socio-Economic Aspects of Fisheries in Inland and Coastal Waters of the Western Balkan. Ecological Sustainability of Fish Resources of Inland Waters of the Western Balkans: Freshwater Fish Stocks, Sustainable Use and Conservation.

[19] Prifti V. (2016). Artificial fertilization of the Ohrid trout and the presence of its summer form in the lake. Sci. Pap. Ser. D. Anim. S..

[20] Spirkovski Z. Changes in the spawning ecology of the Lake Ohrid trout, *Salmo letnica* (Karaman). Proceedings of the 2nd Congress of Ecologists of the Republic of Macedonia with International Participation. Special issues of Macedonian Ecological Society.

[21] Spirkovski Z., Ilik-Boeva D., Ritterbusch D., Peveling R., Pietrock M. (2019). Ghost net removal in ancient Lake Ohrid: A pilot study. Fish. Res..

[22] Prifti V., Cake A. (2017). The Impact of Environmental Conditions on Growth and Development of *Salmo letnica* Smolt. Sci. Pap. Anim. Sci. Biotechnol..

[23] Bernatchez L., Guyomard R., Bonhomme F. (1992). DNA sequence variation of the mitochondrial control region among geographically and morphologically remote European brown trout *Salmo trutta* populations. Mol. Ecol..

[24] Apostolidis A.P., Karakousis Y., Triantaphyllidis C. (1996). Genetic differentiation and phylogenetic relationships among Greek *Salmo trutta* L. (brown trout) populations as revealed by RFLP analysis of PCR amplified mitochondrial DNA segments. Heredity.

[25] Apostolidis A.P., Triantaphyllidis C., Kouvatsi A., Economidis P.S. (1997). Mitochondrial DNA sequence variation and phylogeography among *Salmo trutta* L. (Greek brown trout) populations. Mol. Ecol..

[26] Sušnik S., Snoj A., Wilson I.F., Mrdak D., Weiss S. (2007). Historical demography of brown trout (*Salmo trutta*) in the Adriatic drainage including the putative *S. letnica* endemic to Lake Ohrid. Mol. Phylogenet. Evol..

[27] Snoj A., Marić S., Berrebi P., Crivelli A.J., Shumka S., Sušnik S. (2009). Genetic architecture of trout from Albania as revealed by mtDNA control region variation. Genet. Sel. Evol..

[28] Segherloo I.H., Freyhof J., Berrebi P., Ferchaud A.L., Geiger M., Laroche J., Levin B.A., Normandeau E., Bernatchez L. (2021). A genomic perspective on an old question: *Salmo* trouts or *Salmo trutta* (Teleostei: Salmonidae)?. Mol. Phylogenet. Evol..

[29] Wan Q.H., Wu H., Fujihara T., Fang S.G. (2004). Which genetic marker for which conservation genetics issue?. Electrophoresis.

[30] Galtier N., Nabholz B., Glémin S., Hurst G.D. (2009). Mitochondrial DNA as a marker of molecular diversity: A reappraisal. Mol. Ecol..

[31] Ford M., Salmo letnica The IUCN Red List of Threatened Species 2024, e.T19858A137328386. https://www.iucnredlist.org/species/19858/137328386.

[32] Milana V., Ciampoli M., Sola L. (2014). mtDNA sequences of *Sphyraena viridensis* (Perciformes: Sphyraenidae) from Italy: Insights into historical events and the phylogeny of the genus. Biol. J. Linn. Soc..

[33] Okonechnikov K., Golosova O., Fursov M., The UGENE Team (2012). Unipro UGENE: A unified bioinformatics toolkit. Bioinformatics.

[34] Snoj A., Melkič E., Sušnik S., Muhamedagić S., Dovč P. (2002). DNA phylogeny supports revised classification of *Salmothymus obtusirostris*. Biol. J. Linn. Soc..

[35] Sušnik S., Schöffmann J., Snoj A. (2004). Phylogenetic position of Salmo (Platysalmo) platycephalus Behnke 1968 from south-central Turkey, evidenced by genetic data. J. Fish Biol..

[36] Grapci-Kotori L., Vavalidis T., Zogaris D., Šanda R., Vukić J., Geci D., Ibrahimi H., Bilalli A., Zogaris S. (2020). Fish distribution patterns in the White Drin (Drini i Bardhë) river, Kosovo. Knowl. Manag. Aquat. Ecosyst..

[37] Duftner N., Weiss S., Medgyesy N., Sturmbauer C. (2003). Enhanced phylogeographic information about Austrian brown trout populations derived from complete mitochondrial control region sequences. J. Fish Biol..

[38] Cortey M., García-Marín J.L. (2002). Evidence for phylogeographically informative sequence variation in the mitochondrial control region of Atlantic brown trout. J. Fish Biol..

[39] Cortey M., Pla C., García-Marín J.L. (2004). Historical biogeography of Mediterranean trout. Mol. Phylogenet. Evol..

[40] Meraner A., Baric S., Pelster B., Dalla Via J. (2007). Trout (*Salmo trutta*) mitochondrial DNA polymorphism in the centre of the marble trout distribution area. Hydrobiologia.

[41] Splendiani A., Fioravanti T., Giovannotti M., Olivieri L., Ruggeri P., Nisi Cerioni P., Vanni S., Enrichetti F., Caputo Barucchi V. (2017). Museum samples could help to reconstruct the original distribution of Salmo trutta complex in Italy. J. Fish Biol..

[42] Librado P., Rozas J. (2009). DnaSP v5: A software for comprehensive analysis of DNA polymorphism data. Bioinformatics.

[43] Darriba D., Taboada G.L., Doallo R., Posada D. (2012). jModelTest 2: More models, new heuristics and parallel computing. Nat. Methods.

[44] Ronquist F., Teslenko M., Van der Mark P., Ayres D.L., Darling A., Höhna S., Larget B., Liu L., Suchard M.A., Huelsenbeck J.P. (2012). MrBayes 3.2: Efficient bayesian phylogenetic inference and model choice across a large model space. Syst. Biol..

[45] Gelman A., Rubin D.B. (1992). Inference from iterative simulation using multiple sequences. Stat. Sci..

[46] Peakall R., Smouse P.E. (2012). GenAlEx 6.5: Genetic analysis in excel. Population genetic software for teaching and research—An update. Bioinformatics.

[47] Bandelt H.J., Forster P., Rohl A. (1999). Median-joining networks for inferring intraspecific phylogenies. Mol. Biol. Evol..

[48] Hashem S., Kawai K., Fatsi P.S.K., Kodama A., Saito H. (2020). Genetic relationships of cichlid fishes from Lake Malawi based on mitochondrial DNA sequences. Limnology.

[49] Karaman S. (1957). The Radika river trouts. Folia Balc..

[50] Wilson A.B., Glaubrecht M., Meyer A. (2004). Ancient lakes as evolutionary reservoirs: Evidence from the thalassoid gastropods of Lake Tanganyika. Proc. R. Soc. Lond. B..

[51] Albrecht C., Trajanovski S., Kuhn K., Streit B., Wilke T. (2006). Rapid evolution of an ancient lake species flock: Freshwater limpets (Gastropoda: Ancylidae) in the Balkan lake Ohrid. Org. Divers. Evol..

[52] Radoman P. (1985). Hydrobioidea a superfamily Prosobranchia (Gastropoda), II. Origin, Zoogeography, Evolution in the Balkans and Asia Minor. Monographs Institute of Zoology.

[53] Baillie S.M., Muir A.M., Hansen M.J., Krueger C.C., Bentzen P. (2016). Genetic and phenotypic variation along an ecological gradient in lake trout *Salvelinus namaycush*. BMC Evol. Biol..

[54] Bianco P.G. (2014). An update on the status of native and exotic freshwater fishes of Italy. J. Appl. Ichthyol..

[55] Marić S., Sušnik Bajec S., Schöffmann J., Kostov V., Snoj A. (2017). Phylogeography of stream-dwelling trout in the Republic of Macedonia and a molecular genetic basis for revision of the taxonomy proposed by *S*. karaman. Hydrobiologia.

[56] Ivanova-Davidovic J., Cingoski V., Pavleski V., Saveski V. Upgrading of the Spilje HPP. Proceedings of the 13th Seminar on Hydropower Plants.

[57] Sanna D., Scarpa F., Lai T., Cossu P., Falautano M., Castriota L., Andaloro F., Follesa M.C., Francalacci P., Curini-Galletti M. (2015). *Fistularia commersonii* (Teleostea: Fistulariidae): Walking through the Lessepsian paradox of mitochondrial DNA. Ital. J. Zool..

[58] Sanna D., Azzena I., Scarpa F., Cossu P., Pira A., Gagliardi F., Casu M. (2021). First record of the alien species *Procambarus virginalis* Lyko, 2017 in freshwaters of Sardinia and insight into its genetic variability. Life.

[59] Locci C., Azzena I., Pascale N., Ciccozzi A., Deplano I., Giantsis I.A., Papadopoulos D.K., Lattos A., Orrù F., Puzzi C.M. (2024). A sister species for the blue crab, *Callinectes sapidus*? A tale revealed by mitochondrial DNA. Life.

[60] Pennell M.W., Harmon L.J., Uyeda J.C. (2014). Is there room for punctuated equilibrium in macroevolution?. Trends Ecol. Evol..

[61] Eldredge N., Gould S., Schopf Tomas J.M. (1972). Punctuated equilibria: An alternative to phyletic gradualism. Models in Paleobiology.

