# Supplementary figures and images for: Exploring Mitochondrial Evolutionary Pathways: Insights into the Origin of the Endemic Ohrid Trout
Source: Life (Basel). 2025 Jan 3;15(1):52. doi: 10.3390/life15010052 (PMC11766461; doi:10.3390/life15010052)

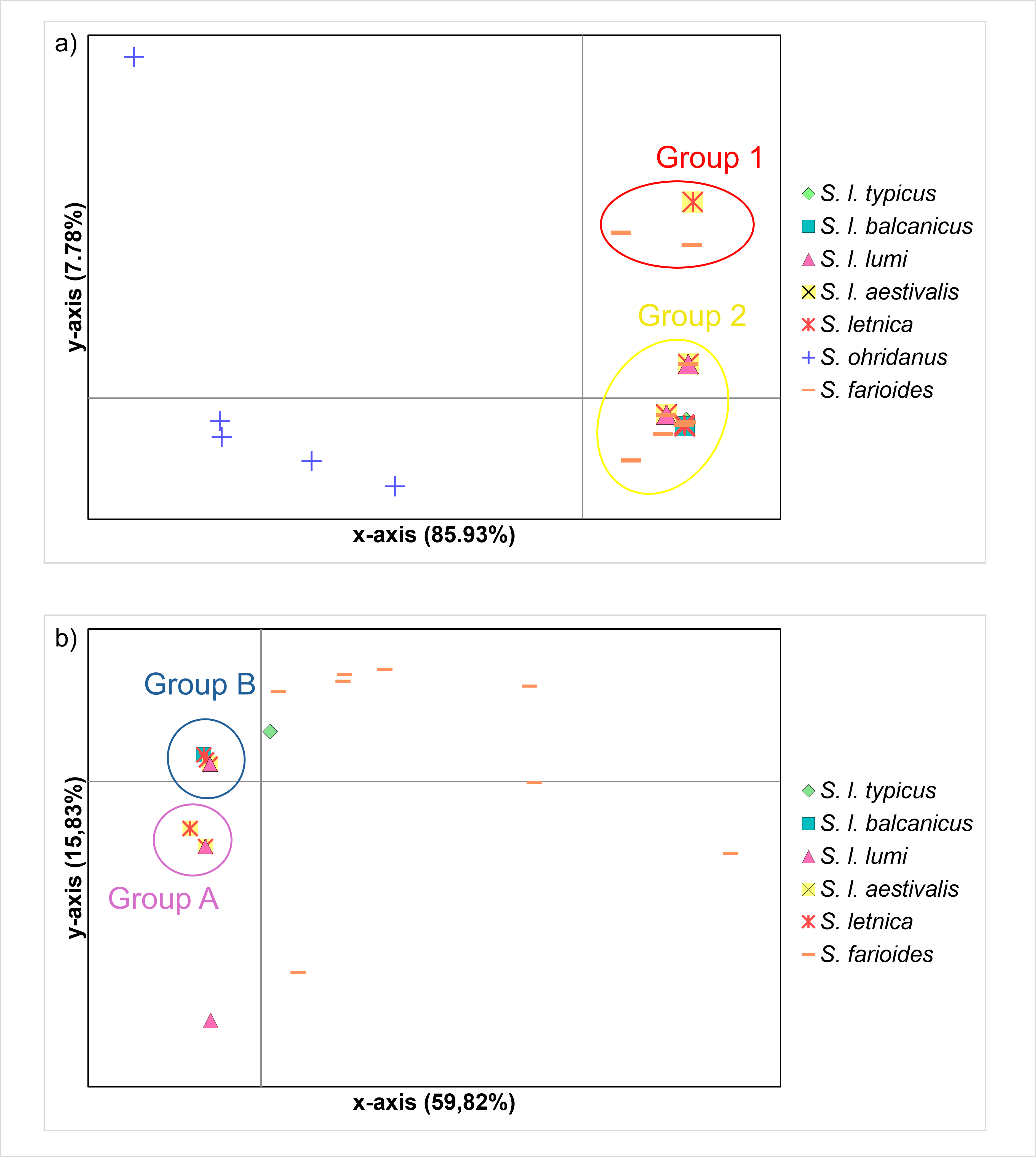

Supplement: Supplementary file 1 [file life-15-00052-s001.zip › Supplementary Figure S1.tif]
